# CXCL-8 in Preoperative Colorectal Cancer Patients: Significance for Diagnosis and Cancer Progression

**DOI:** 10.3390/ijms21062040

**Published:** 2020-03-17

**Authors:** Sara Pączek, Marta Łukaszewicz-Zając, Mariusz Gryko, Piotr Mroczko, Agnieszka Kulczyńska-Przybik, Barbara Mroczko

**Affiliations:** 1 Department of Biochemical Diagnostics, Medical University of Bialystok, 15-269 Bialystok, Poland; sara.paczek@umb.edu.pl (S.P.); mroczko@umb.edu.pl (B.M.); 2 Second Department of General Surgery, Medical University of Bialystok, 15-269 Bialystok, Poland; mariusz_gryko@vp.pl; 3 Department of Criminal Law and Criminology, Faculty of Law, University of Bialystok, 15-213 Bialystok, Poland; p.mroczko@uwb.edu.pl; 4 Department of Neurodegeneration Diagnostics, Medical University of Bialystok, 15-269 Bialystok, Poland; agnieszka.kulczynska-przybik@umb.edu.pl

**Keywords:** cancer progression, tumor markers, prognostic biomarkers

## Abstract

**Introduction.** Since colorectal cancer (CRC) is the second most commonly diagnosed malignancy in Europe and third worldwide, novel biomarkers for diagnosing the disease are critically needed. **Objectives.** According to our knowledge, the present study is the first to evaluate the clinical usefulness of serum CXCL-8 (C-X-C motif chemokine 8) in the diagnosis and progression of CRC compared to classical tumor marker CEA (carcinoembryonic antigen) and marker of inflammation CRP (C-reactive protein). **Patients and Methods.** The study included 59 CRC patients and 46 healthy volunteers. Serum levels of selected proteins were measured using ELISA (enzyme-linked immunosorbent assay), CMIA (chemiluminescent microparticle immunoassay), and immunoturbidimetric methods. **Results.** Serum concentrations of CXCL-8, similarly to those of the classical tumor marker CEA and inflammatory state marker CRP, were significantly higher in CRC patients than in healthy controls. There were statistically significant differences in CXCL-8 concentrations between tumor stages, as established by the Kruskal–Wallis test and confirmed by the post hoc Dwass–Steele–Critchlow–Fligner test. CXCL-8 levels were also significantly elevated in CRC patients with distant metastases compared to patients in the subgroup without metastases. Diagnostic sensitivity, predictive values for negative results (NPV), and AUC (area under the Receiver Operating Characteristic Curve—ROC curve) of CXCL-8 were higher than those of CEA, while diagnostic specificity and predictive values for positive results (PPV) of CXCL-8 were higher than those of CRP. **Conclusions.** Our findings indicate greater utility of CXCL-8 in comparison to the classical tumor marker CEA in the diagnosis of CRC. Moreover, serum CXCL-8 might be a potential biomarker of colorectal cancer progression.

## 1. Introduction

Colorectal cancer (CRC) is a heterogeneous disease occurring either in the colon or the rectum [1]. Despite a relatively low incidence of CRC several years ago, a rising incidence, as well as increasing mortality due to other lifestyle factors, have been observed recently [2]. Almost 150,000 new cases of CRC were estimated to be diagnosed in the USA alone in 2019. CRC is the third most common cause of death for both men and women worldwide [3]. Despite widespread screening programs, the late diagnosis of CRC continues to be a global problem [4]. It has been demonstrated that CRC incidence is strongly influenced by nutrition, with an unhealthy high fat/high carbohydrate Western-style diet significantly contributing to an increase in the number of CRC cases [5].

CRC can be divided into two types: hereditary and non-hereditary. The majority of CRC cases are non-hereditary, caused mainly by somatic mutations in response to environmental factors [1]. Colorectal carcinogenesis is normally a lengthy and very complex process comprising several steps of malignant transformation from normal epithelium to cancer cells and typically involving numerous genetic changes that result in various phenotypic alterations [6]. It has been suggested that selected chemokines are involved in a number of processes, including those relating to CRC, leading to cancerous transformation [7].

Chemokines are small, soluble chemotactic cytokines that are able to bind to their cognate G-protein-coupled receptors, thereby stimulating the migration, adhesion or chemotaxis of cells [8]. Structurally, chemokines are divided into four subgroups according to the arrangement of *N*-terminal cysteine residues (CXC, CX3C, CC, and C) [9]. A number of investigations have indicated that these proteins may facilitate tumor progression, which has made them the focus of research efforts.

CXCL-8 (C-X-C motif chemokine 8), which is also known as IL-8 (interleukin-8), is mainly produced by macrophages, and, therefore, it is primarily responsible for the chemotaxis of neutrophils towards the site of an inflammatory process [10]. It has been suggested that this protein may play a role in tumor progression, chiefly by regulating the process of angiogenesis, tumor growth, and proliferation as well as the survival of malignant cells. A number of studies have demonstrated CXCL-8 expression on endothelial cells, tumor-associated macrophages, and cancer cells, including CRC [11]. Furthermore, some authors have indicated that CXCL-8 overexpression is correlated with the presence of distant metastases and general tumor progression, which may indicate the potential usefulness of this protein as a marker of progression of this disease [12].

The diagnosis of CRC is based on invasive methods such as colonoscopy and sigmoidoscopy. A non-invasive alternative is the assessment of serum CEA concentration. However, due to its lack of diagnostic sensitivity and specificity, novel blood biomarkers useful in the early detection of the disease are critically needed [8,13].

The current study is a continuation of our previous investigations concerning the diagnostic significance of selected chemokines and their specific receptors in gastrointestinal tumors, including esophageal and pancreatic cancer, where we assessed the potential usefulness of serum levels of these molecules as potential tumor markers for the malignancies mentioned above [14,15,16,17].

Therefore, the aim of the present study is to investigate whether chemokine CXCL-8 may be used as a potential biochemical tumor marker for CRC. Therefore, we examined serum levels of CXCL-8 in relation to tumor stage, and clinicopathological parameters of CRC in order to evaluate its significance in CRC progression. Moreover, using diagnostic criteria, we assessed the diagnostic usefulness of this chemokine in relation to the classical tumor marker CEA and a marker of inflammation C-reactive protein (CRP). According to our knowledge, the present study is the first to assess the clinical usefulness of serum CXCL-8 (C-X-C motif chemokine 8) in the diagnosis and progression of CRC compared to the classical tumor marker CEA (carcinoembryonic antigen) and the marker of inflammation CRP (C-reactive protein).

## 2. Patients and Methods

The total study group comprised 105 patients—59 patients with colorectal cancer (29 females and 30 males) and 46 healthy volunteers (21 females and 25 males). All cancer patients were diagnosed and underwent surgery in the Second Department of General Surgery, Medical University in Bialystok (Poland). Clinical diagnosis of CRC was based on the histopathological examination of tissue samples. The colorectal cancer stage was determined according to the TNM (tumor–nodes–metastasis) staging system created by UICC (International Union Against Cancer). Furthermore, the CRC group was divided into subgroups depending on the depth of tumor invasion (T-factor), presence of lymph node metastasis (N-factor), and distant metastasis (M-factor), as well as tumor stage (TNM). The characteristics of the study group are presented in Table 1.

All study participants provided written informed consent and the project was approved by the Local Ethics Committee (R-I-002/65/2017) of the Medical University of Bialystok (Poland).

Blood samples were collected from CRC patients prior to treatment and stored frozen at −80 °C until assessment. Serum CXCL-8 concentration was measured using ELISA (enzyme-linked immunosorbent assay) kit (Quantikine ELISA Human CXCL-8/IL-8 Immunoassay, Abingdon, R&D Systems, UK) in accordance with the manufacturer’s instructions. Serum levels of the classical tumor marker CEA (carcinoembryonic antigen) were assessed using the CMIA (chemiluminescent microparticle immunoassay) method on ARCHITECT 8200 ci (Abbott Laboratories, Abbott Park, IL, USA). Serum CRP (C-reactive protein) concentrations were measured by means of the immunoturbidimetric method on ARCHITECT 8200 ci (Abbott Laboratories, Abbott Park, IL, USA).

### Statistical Analysis

Serum concentrations of CXCL-8, CEA, and CRP did not follow a normal distribution in the preliminary statistical analysis (χ2-test). Therefore, nonparametric statistical analyses were utilized. The Mann–Whitney test was used to compare two groups, whereas the Kruskal–Wallis test was employed to analyze three or more groups. Moreover, the post hoc Dwass–Steele–Critchlow–Fligner test was performed on parameters displaying statistically significant differences. Correlation analyses were conducted using the Spearman’s rank correlation test. Furthermore, diagnostic parameters such as diagnostic sensitivity and specificity, accuracy, predictive values for positive (PPV) and negative (NPV) results as well as area under the ROC curve (AUC) for the tested proteins were also evaluated. Differences were considered statically significant when *p* < 0.05. CXCL-8 concentrations below the limit of detection were analyzed as zero values. For statistical analysis, IBM SPSS Statistics 20.0 (New York, NY, USA) was employed, while diagnostic parameters were calculated using Microsoft Office Excel (New York, NY, USA) To assess correlations between risk factors and CRC, logistic regression was used. Initially, univariate logistic regression models were obtained for each risk factor and, subsequently, for variables, *p* < 0.05 multivariate analyses were employed. In addition, the stripcharts with boxplots were created using an R-studio program (version 1.2.5019) (Boston, MA, USA). 

Youden’s index was used to select the optimal predicted probability cut-off values. The reference cut-off values were 12.57 ng/mL for CXCL-8, 1.45 mg/L for CRP, and 3.55 ng/mL for CEA.

## 3. Results

### 3.1. Serum Levels of CXCL-8, CEA, and CRP in Colorectal Cancer Patients

**Figure 1:**
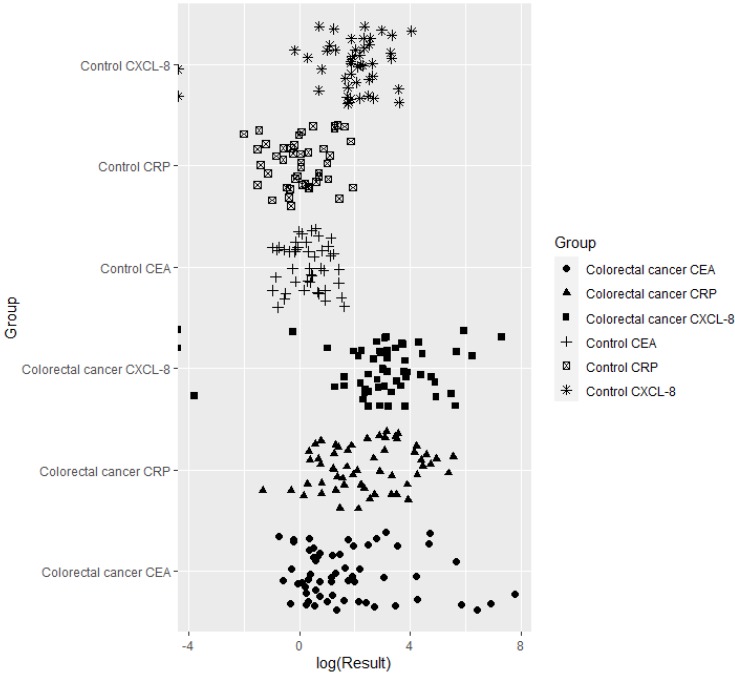
The logarithmic dependence of serum CXCL-8, CEA and CRP concentrations in CRC patients in comparison to healthy controls.

Serum concentrations, including the ranges and medians, of CXCL-8, as well as those of the classical tumor marker CEA (carcinoembryonic antigen) and a marker of inflammatory status, CRP (C-reactive protein), are presented in Table 2 and Figure 1. Serum CXCL-8 levels were significantly higher (*p* < 0.001) in CRC patients in comparison to healthy volunteers. Similar, statistically significantly higher, results were obtained for serum concentrations of CEA (*p* < 0.001) and CRP (*p* < 0.001) (Table 2, Figure 1).

**Figure 2:**
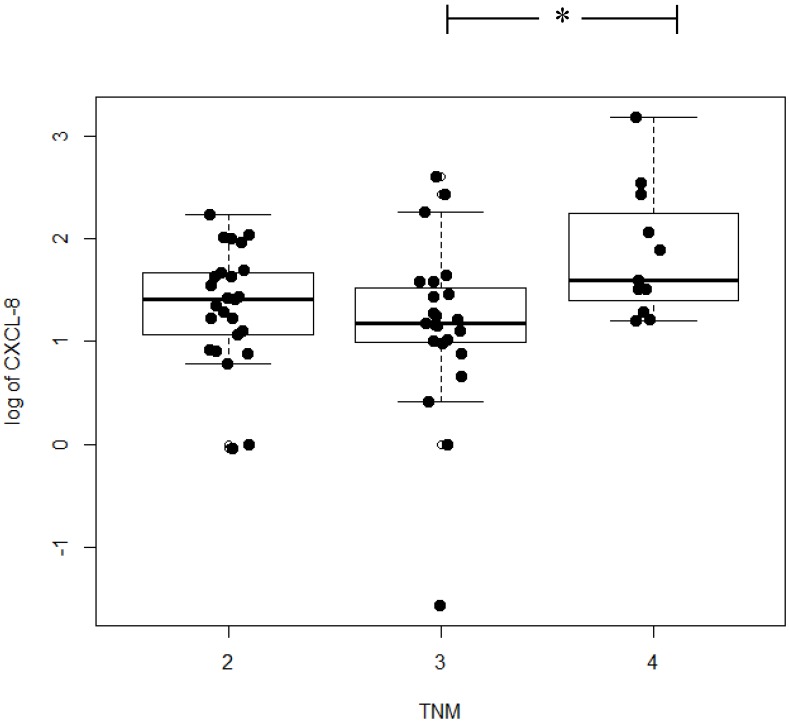
Serum concentrations of chemokine CXCL-8 in colorectal cancer patients according to TNM classification. * Statistically significant.

If we consider the relationship between the examined proteins and tumor stage, the highest concentrations of CXCL-8, CEA, and CRP were found in patients with stage IV cancer. Statistically significant differences in CXCL-8 concentrations between tumor stages were first established using the Kruskal–Wallis test (*p* = 0.029; Figure 2) and then confirmed by the post hoc Dwass–Steele–Critchlow–Fligner test in patients with stage III and IV of CRC (*p* = 0.021; data not shown). Similar results were obtained for CEA (*p* = 0.001) and the marker of inflammation, CRP (*p* = 0.013) (Table 3).

### 3.2. Relationship Between Serum Levels of CXCL-8, CEA, and CRP in CRC Patients and Clinicopathological Features of CRC

Having analyzed associations between serum concentrations of the tested proteins and the depth of tumor invasion (T-factor) in CRC, we found that serum levels of CXCL-8 were highest in the T_4_ subgroup, but the differences between the subgroups (T_1_ + T_2_, T_3_, and T_4_) were not significant. The concentrations of CEA and CRP were also highest in the T_4_ subgroup and, similarly to CXCL-8, were not statistically significant (Table 3).

As for the presence of lymph node metastasis (N-factor), serum concentrations of CXCL-8 were higher in the N_1_ subgroup in comparison to patients without nodal involvement (N_0_ subgroup), but the differences were not significant. Statistical differences between the N_0_ and N_1_ subgroups were found only for serum levels of CEA (*p* = 0.005; Table 3).

**Figure 3:**
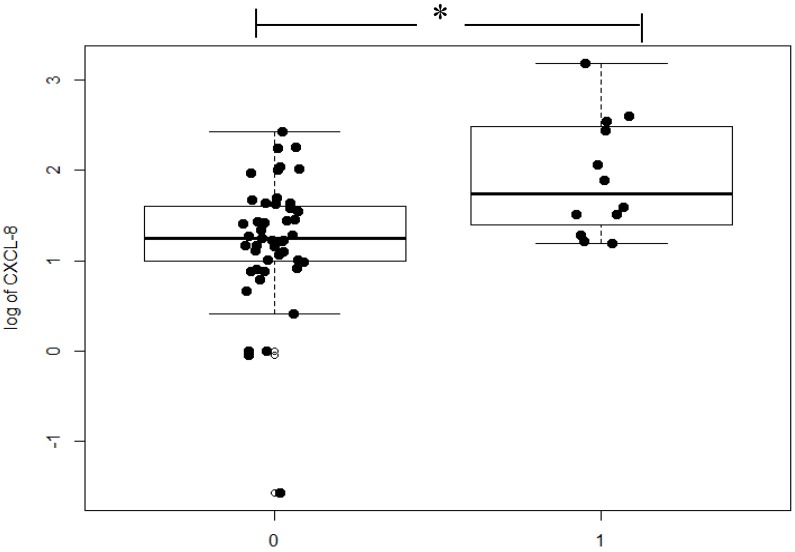
Serum concentrations of chemokine CXCL-8 in groups of patients with distant metastases (M1) and without distant metastases (M0). * Statistically significant.

If we consider the presence of distant metastases (M-factor), serum CXCL-8 concentration was found to be significantly higher in the M_1_ subgroup in comparison to patients without distant metastases (*p* = 0.004; Figure 3). As for the remaining proteins tested, elevated serum CEA (*p* < 0.001) and CRP (*p* = 0.009) concentrations were also observed to be significant.

Correlations between concentrations of the tested proteins and clinicopathological features of the tumor were assessed using the Spearman’s rank correlation test (Table 4). Serum concentrations of CXCL-8 were significantly correlated with the presence of distant metastases (*p* = 0.003) as well as with serum levels of CEA (*p* = 0.019) and CRP (*p* < 0.001) in CRC patients (Table 4).The relationship between several risk factors and CRC risk was initially examined using univariate analysis in order to identify the risk factors which qualified for the multivariate model (results are presented as odds ratios (OR) and *p*-values). Only serum concentrations of CRP (*p* = 0.001, OR = 2.018) were found to be associated with a significantly increased risk of CRC occurrence. Therefore, variables that were statistically significant in the univariate logistic regression model were entered into the multivariate analysis. Other, nonsignificant variables were removed from the model. Therefore, in the final analysis, only serum concentrations of CRP and CEA (*p* = 0.001, OR = 2.033; *p* = 0.046, OR = 1.545) were indicated to be significant risk factors of CRC occurrence.

### 3.3. Diagnostic Usefulness of CXCL-8 in CRC Diagnosis

**Figure 4:**
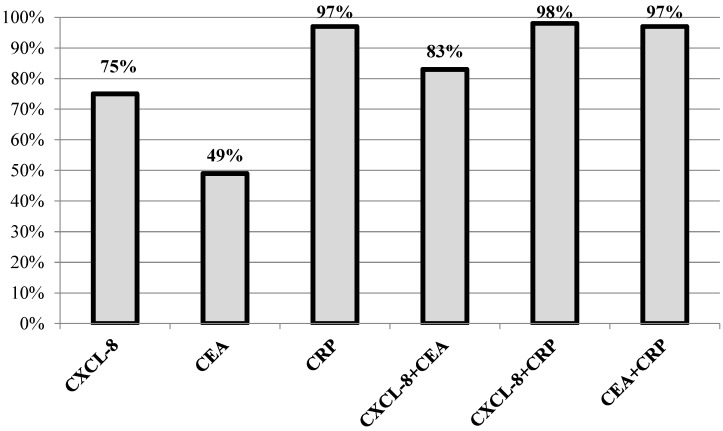
Diagnostic sensitivity for the measurement of tested proteins.

**Figure 5:**
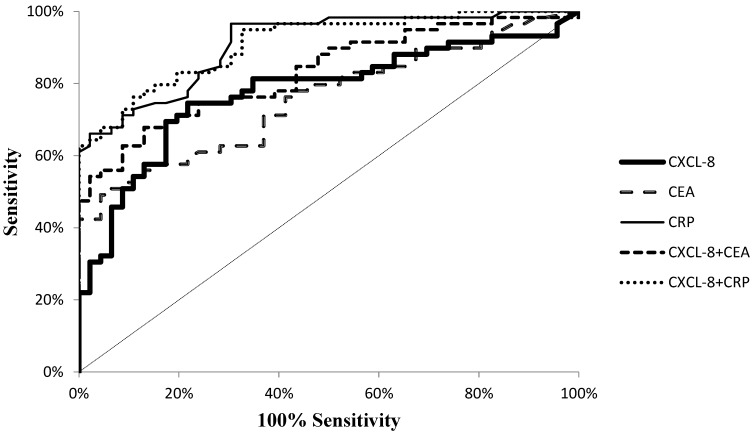
Receiver Operating Characteristic Curve (ROC) curve for analyzed proteins.

Diagnostic characteristics such as diagnostic sensitivity, diagnostic specificity, accuracy, predictive values for positive (PPV) and negative (NPV) results as well as the areas under the ROC curve (AUC) were calculated to assess the potential usefulness of CXCL-8 in the diagnosis of CRC. We observed that the diagnostic sensitivity (the percentage of elevated results) of CXCL-8 was higher (75%) than that of CEA (49%) but lower than that of CRP (97%) (Figure 4). Moreover, the highest diagnostic sensitivity was obtained for the combined analysis of CXCL-8 and CRP (98%; Figure 4). The same dependency was observed for negative predictive value (NPV), which was higher for CXCL-8 (71%) in comparison to CEA (60%) and lower in comparison to CRP (94%). Furthermore, the diagnostic specificity of the tested proteins was also assessed. We observed that the diagnostic specificity was higher for CXCL-8 (78%) than for CRP (70%), but the highest specificity was calculated for CEA concentrations (96%). The diagnostic accuracy (ACC) of CXCL-8 (76%) was found to be higher than that of CEA (70%), but lower than that of CRP (85%). The area under the ROC curve (AUC) signifies the clinical usefulness of studied proteins in diagnosing disease. The AUC for CXCL-8 (0.7775; *p* < 0.001) was higher than that for CEA (0.7579; *p* < 0.001), but lower than that for CRP (0.9101; *p* < 0.001) in CRC diagnosis (Figure 5).

## 4. Discussion

Colorectal cancer is considered a serious global problem since it is the third most frequently diagnosed malignancy worldwide. Improving early diagnosis of CRC has therefore been the focus of investigation efforts in recent years. Any medical activities performed by a doctor must be conducted in accordance with medical law. The aim of these regulations is to protect the rights of the patient, as well as to establish certain principles to be followed by the doctor. The specific regulations may vary depending on where the medical procedure is performed. However, there are two principles that are common to the laws of different countries. The first rule of medical law, which concerns, inter alia, the treatment of a patient with cancer, is the obligation to inform the patient about his or her condition. Finding the new substances, e.g., CXCL-8, whose determination could recognize the presence of cancer more precisely than previously known biochemical compounds, would give the doctor the opportunity to provide the patient with more reliable information about his or her condition and thus will help to make a better therapeutic decision. This is particularly important in view of the second principle, according to which the patient has the right to decide on his/her treatment process. This means that, in general, the patient’s consent is required to carry out the medical procedure. It is crucial in the case of cancer when the quality of the decision to choose an effective treatment method is influenced by a reliable diagnosis [18,19].

Many investigations have indicated that selected chemokines may be involved in tumor development as well as in supporting the formation of the metastatic spread [20]. A prime example is chemokine CXCL-8, which has demonstrated to be involved in tumor angiogenesis and linked with promoting distant metastases in many malignancies, including CRC [21].

This paper is a continuation of our previous studies investigating the utility of specific proteins, such as chemokines, as potential tumor biomarkers for gastrointestinal malignancies [22,23,24]. Despite an increasing number of investigations outlining the multifunctional role of CXCL-8 in CRC progression, our study is the first to evaluate the significance of CXCL-8 as a potential biomarker of CRC in comparison to the classical tumor marker.

Our study revealed that serum levels of CXCL-8, similarly to those of the classical tumor marker and CRP, were significantly higher in CRC patients in comparison to the healthy controls. The same dependency was observed in a study by Sgourakis et al. [25], who demonstrated that CXCL-8 concentrations were higher in the blood of CRC patients in comparison to the healthy controls. Furthermore, based on immunohistochemical methods, Oladipo et al. have demonstrated that the majority of tumor cores present positive CXCL-8 expression within tumor-associated inflammatory infiltrate [26]. A similar observation was made by Rubie et al., who proved that CXCL-8 expression was significantly higher in all CRC tissue in comparison to inflammatory and non-malignant samples [27]. In our previous studies, we evaluated serum CXCL-8 concentrations in gastrointestinal malignancies and revealed that serum concentrations of CXCL-8, similarly to those of the classical tumor markers (SCC-Ag and CEA) are significantly higher in esophageal cancer (OC) patients in comparison to healthy controls [15]. We have obtained similar results in our studies investigating serum CXCL-8 levels in pancreatic cancer (PC) patients, where significantly elevated CXCL-8 concentrations were revealed in PC patients in comparison to healthy controls [17]. These findings may suggest that CRC cells are able to produce CXCL-8, which might be a non-specific biomarker of CRC.

In the current study, we analyzed the relationship between the tested proteins and the stage of CRC. We demonstrated statistically significant differences in CXCL-8 concentrations between tumor stages using the Kruskal–Wallis test (*p* = 0.029) and confirmed them with the post hoc Dwass–Steele–Critchlow–Fligner test in patients with stage III and IV of CRC (*p* = 0.021; data not shown). Our results are in line with the results obtained by other authors who have revealed that serum CXCL-8 levels are significantly correlated with the CRC stage [28]. Similar results have been obtained using immunohistochemical methods, where the expression of CXCL-8 in CRC tissue was correlated with more advanced stages of the disease [29,30,31]. Therefore, the authors have suggested that the absence of CXCL-8 expression in epithelial cells might be a factor indicating good prognosis [26].

When we analyzed the relationship between serum levels of CXCL-8 and clinicopathological parameters, we discovered that serum CXCL-8 concentrations were significantly higher in CRC patients with distant metastasis in comparison to those without metastasis, which was confirmed by the Spearman rank correlation test. In a study of Ruby et al., the association between CXCL-8 levels and clinicopathological features of CRC was also evaluated [27]. The authors found significant CXCL-8 overexpression in CRC tissues which correlated with tumor size and depth of tumor invasion [29,32].

In the present study, we compared the diagnostic characteristics of CXCL-8 with the classical tumor markers for CRC. Diagnostic sensitivity, predictive value of negative (NPV) results and accuracy were higher for serum concentrations of CXCL-8 in comparison to the classical tumor marker CEA. Furthermore, diagnostic specificity and predictive value of positive (PPV) results were higher for serum levels of CXCL-8 than for CRP. Moreover, the AUC for CXCL-8 was higher than for CEA in CRC patients. Similar findings have been published by other authors who demonstrated that the AUC for CXCL-8 was 0.742, while sensitivity and specificity for CXCL-8 were 85% and 54%, respectively, in CRC patients. If we consider the diagnostic characteristics of the tested proteins, we may conclude that serum CXCL-8 might be a better candidate for a biomarker in the diagnosis of CRC than CEA, which is currently used in routine clinical practice.

## 5. Conclusions

The incidence rate of CRC has increased in the last few years. Therefore, low-cost, non-invasive methods, including the assessment of novel biomarkers useful in early diagnosis, are sorely needed. Many investigations have indicated elevated CXCL-8 expression in CRC patients. However, little is known about the significance of serum CXCL-8 as a potential tumor marker in the diagnosis and progression of CRC. Despite continuing controversy regarding the relevance of CXCL-8 in cancer biology, our study revealed that CXCL-8 is a better biomarker than the currently-used classical tumor marker CEA in the diagnosis of CRC. Moreover, findings presented in this paper confirm the significance of CXCL-8 in CRC progression, particularly in establishing tumor stage and distant metastases. However, due to the non-specific nature of chemokines and relatively low number of subjects included in the study and control group, further investigations are needed to clarify their potential use as biomarkers of CRC.

## Figures and Tables

**Table 1:** Characteristics of the colorectal cancer (CRC) patients group.

Colorectal Cancer Group		Number of Patients
Gender	Male	30
	Female	29
TNM stage	I + II	25
	III	23
	IV	11
Depth of tumor invasion (T-factor)	T1 + T2	6
	T3	47
	T4	6
Lymph node metastases (N-factor)	N0	26
	N1 + N2	33
Distant metastases (M-factor)	M0	47
	M1	12

Abbreviations: TNM, tumor (T), nodes (N), and metastases (M).

**Table 2:** Concentrations of CXCL-8, CEA, and CRP in sera of patients with CRC in comparison to healthy controls.

		CXCL-8 [pg/mL]	CRP [mg/L]	CEA [ng/mL]
Control group *n* = 46	Minimum	0.000	0.50	0.2
	Median	8.61450	1.2150	1.050
	Maximum	72.312	4.54	5.0
Colorectal cancer group *n* = 59	Minimum	0.000	0.50	0.4
	Median	22.18600	3.2000	8.800
	Maximum	1540.375	1500.00	202.3
*p* (Mann–Whitney test)		***p* < 0.001**	***p* < 0.001**	***p* < 0.001**

Abbreviations: CEA, carcinoembryonic antigen; CRP, C-reactive protein; CXCL-8, C-X-C motif chemokine 8; TNM, tumor (T), nodes (N), and metastases (M). Statistically significant when *p* < 0.05.

**Table 3:** Serum concentrations of testes proteins in relation to clinicopathological features.

Colorectal Cancer Group			CXCL-8 (pg/mL)	CRP (mg/L)	CEA (ng/mL)
TNM (Tumor stage)	1 + 2 *n* = 25	Min	0.000	0.4	0.50
		Me	25.43300	9.200	1.6900
		Max	174.082	159.2	118.68
	3 *n* = 23	Min	0.000	1.1	0.69
		Me	14.84200	5.000	3.0700
		Max	401.986	65.2	540.17
	4 *n* = 11	Min	15.703	3.4	1.99
		Me	39.68100	53.700	60.3000
		Max	1540.375	202.3	1500.00
	Kruskal–Wallis test (*p*)		**0.029**	**0.013**	**0.001**
T-factor (The depth of tumor invasion)	T1 *n* = 1	Min	27.255	2.2	3.56
		Me	27.25500	2.200	3.5600
		Max	27.255	2.2	3.56
	T2 *n* = 5	Min	16.686	0.4	0.89
		Me	19.29700	13.600	1.1900
		Max	92.600	57.9	18.92
	T3 *n* = 47	Min	0.000	1.1	0.50
		Me	18.73300	6.100	3.9100
		Max	1540.375	202.3	1500.00
	T4 *n* = 6	Min	14.842	5.3	1.05
		Me	29.49200	32.600	5.9500
		Max	351.515	82.1	1500.00
	Kruskal–Wallis test (*p*)		0.690	0.245	0.515
N-factor (The presence of lymph node metastases)	N0 *n* = 26	Min	0.000	0.4	0.50
		Me	20.76900	11.100	1.7100
		Max	174.082	159.2	118.68
	N1 + N2 *n* = 33	Min	0.000	1.100	0.69
		Me	27.593	8.200	4.820
		Max	1540.375	202.3	1500.00
	Mann–Whitney test (*p*)		0.652	0.737	0.005
M-factor (The presence of distant metastases)	M0 *n* = 47	Min	0.000	0.4	0.50
		Me	17.98700	5.300	2.5900
		Max	270.582	159.2	540.17
	M1 *n* = 12	Min	15.703	3.4	1.99
		Me	58.78200	38.900	65.9850
		Max	1540.375	202.3	1500.00
	Mann–Whitney test (*p*)		**0.004**	**0.009**	***p* < 0.05**

Abbreviations: CEA, carcinoembryonic antigen; CRP, C-reactive protein; CXCL-8, C-X-C motif chemokine 8; TNM, tumor (T), nodes (N), and metastases (M). Statistically significant when *p* < 0.05.

**Table 4:** Correlations between levels of tested proteins and clinicopathological features in the sera of CRC patients.

			T-factor	N-factor	M-factor	TNM	G-factor	Age	CXCL-8	CEA	CRP
CRC group	T-factor	r	1.00	0.25	0.28	0.35	0.01	−0.12	0.08	0.18	0.24
		*p*		0.052	**0.032**	**0.007**	0.951	0.385	0.541	0.173	0.071
	N-factor	r	0.25	1.00	0.35	0.75	−0.15	0.07	0.14	0.42	0.03
		*p*	0.052		**0.007**	**0.000**	0.258	0.604	0.303	**0.001**	0.833
	M-factor	r	0.28	0.35	1.00	0.72	−0.09	0.14	0.38	0.50	0.34
		*p*	**0.032**	**0.007**		**0.000**	0.496	0.300	**0.003**	**0.000**	**0.008**
	TNM	r	0.35	0.75	0.72	1.00	−0.10	0.06	0.15	0.45	0.14
		*p*	**0.007**	**0.000**	**0.000**		0.458	0.646	0.260	**0.000**	0.304
	G-factor	r	0.01	−0.15	−0.09	−0.10	1.00	0.13	−0.06	0.05	−0.01
		*p*	0.951	0.258	0.496	0.458		0.350	0.647	0.731	0.968
	Age	r	−0.12	0.07	0.14	0.06	0.13	1.00	0.09	0.12	0.05
		*p*	0.385	0.604	0.300	0.646	0.350		0.478	0.376	0.707
	CXCL-8	r	0.08	0.14	0.38	0.15	−0.06	0.09	1.00	0.31	0.57
		*p*	0.541	0.303	**0.003**	0.260	0.647	0.478		**0.019**	**0.000**
	CEA	r	0.18	0.42	0.50	0.45	0.05	0.12	0.31	1.00	0.20
		*p*	0.173	**0.001**	**0.000**	**0.000**	0.731	0.376	**0.019**		0.125
	CRP	r	0.24	0.03	0.34	0.14	-0.01	0.05	0.57	0.20	1.00
		*p*	0.071	0.833	**0.008**	0.304	0.968	0.707	**0.000**	0.125	

Abbreviations: CEA, carcinoembryonic antigen; CRC, colorectal cancer; CRP, C-reactive protein; CXCL-8, C-X-C motif chemokine 8; TNM, tumor (T), nodes (N), and metastases (M). Statistically significant when *p* < 0.05.
